# Supplementary figures and images for: Characterization of Coxiella burnetii Dugway Strain Host-Pathogen Interactions In Vivo
Source: Microorganisms. 2022 Nov 15;10(11):2261. doi: 10.3390/microorganisms10112261 (PMC9692954; doi:10.3390/microorganisms10112261)

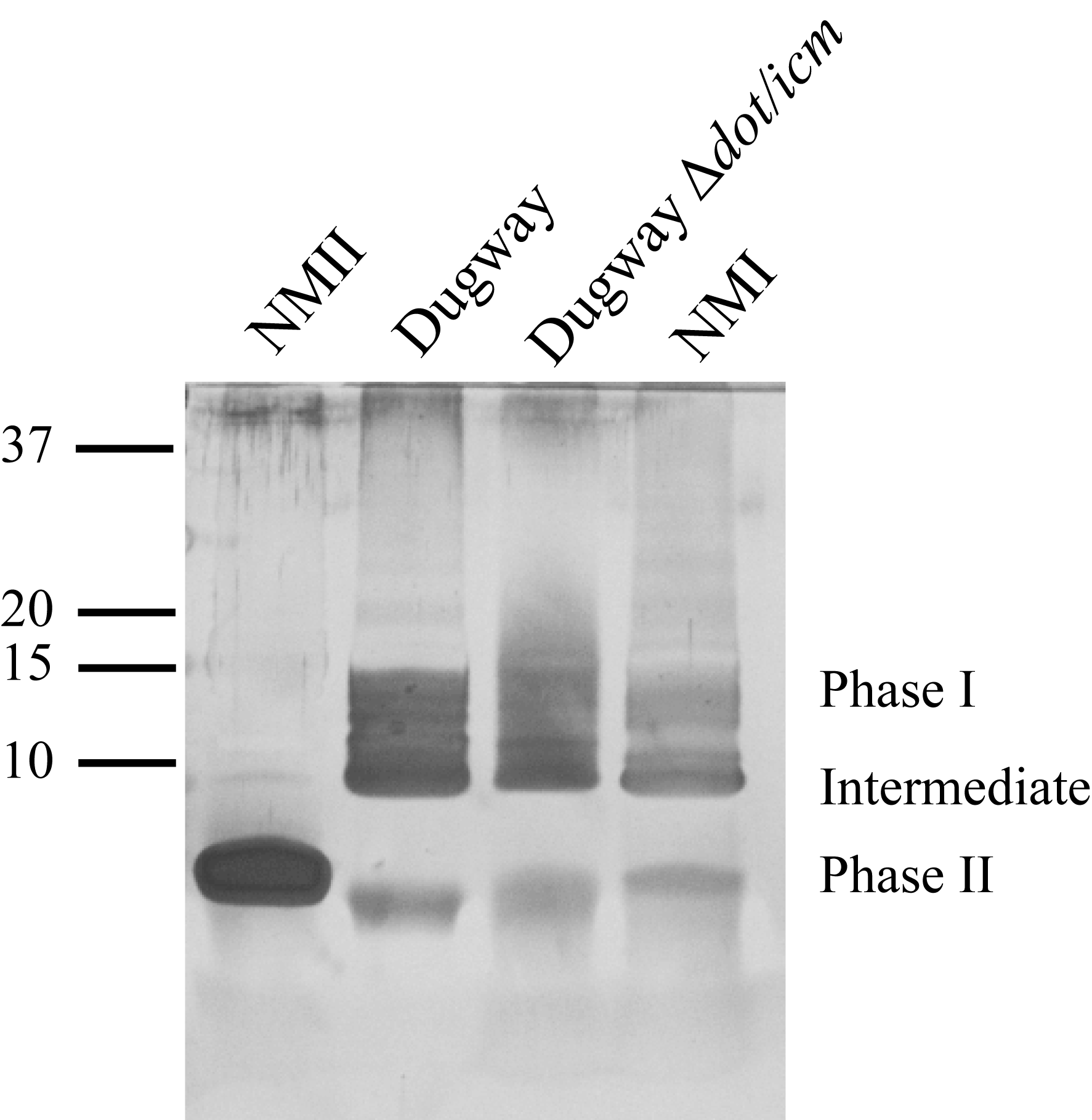

Supplement: Supplementary file 1 [file microorganisms-10-02261-s001.zip › Figure S1.tif]

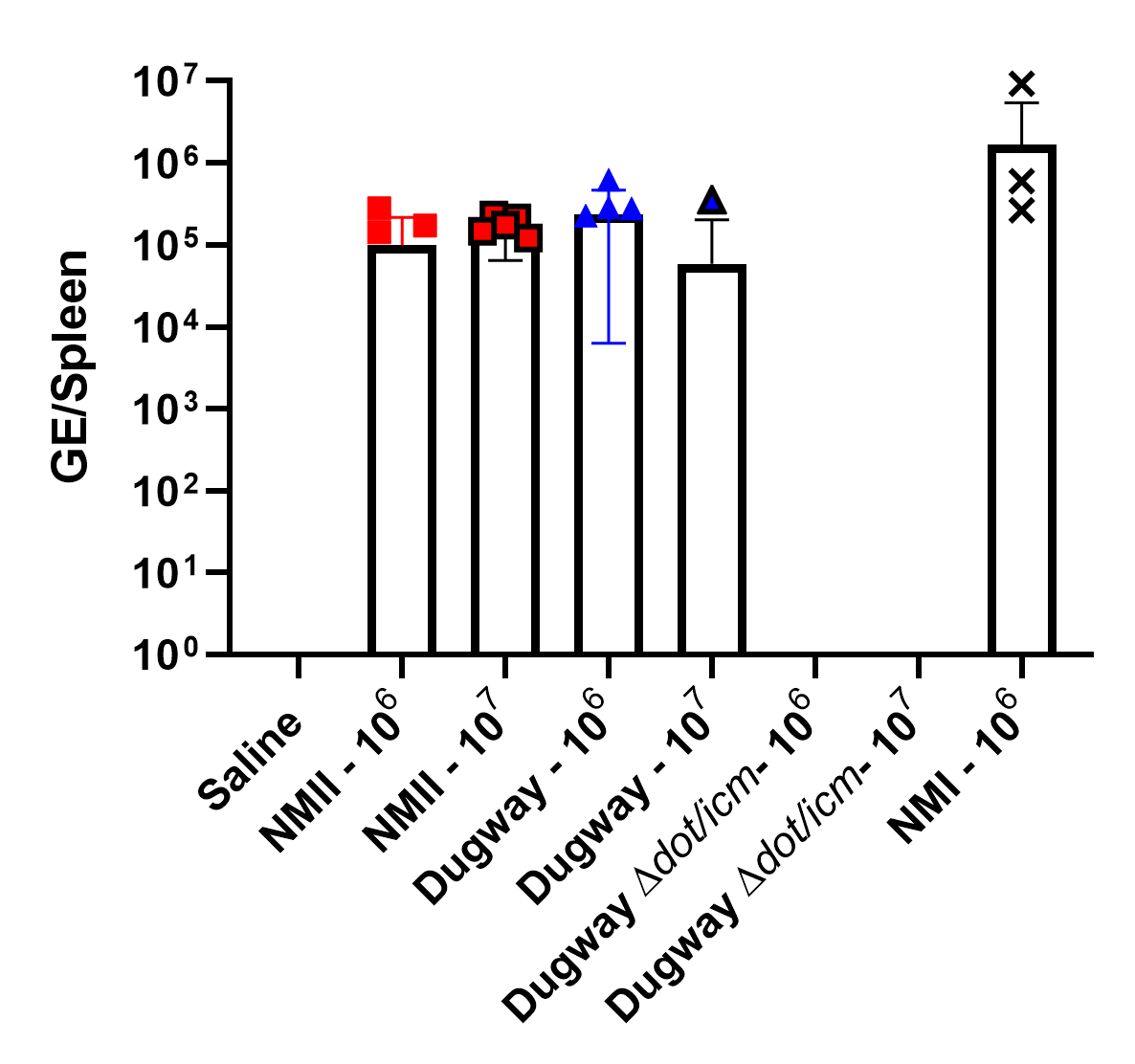

Supplement: Supplementary file 1 [file microorganisms-10-02261-s001.zip › Figure S2.tif]

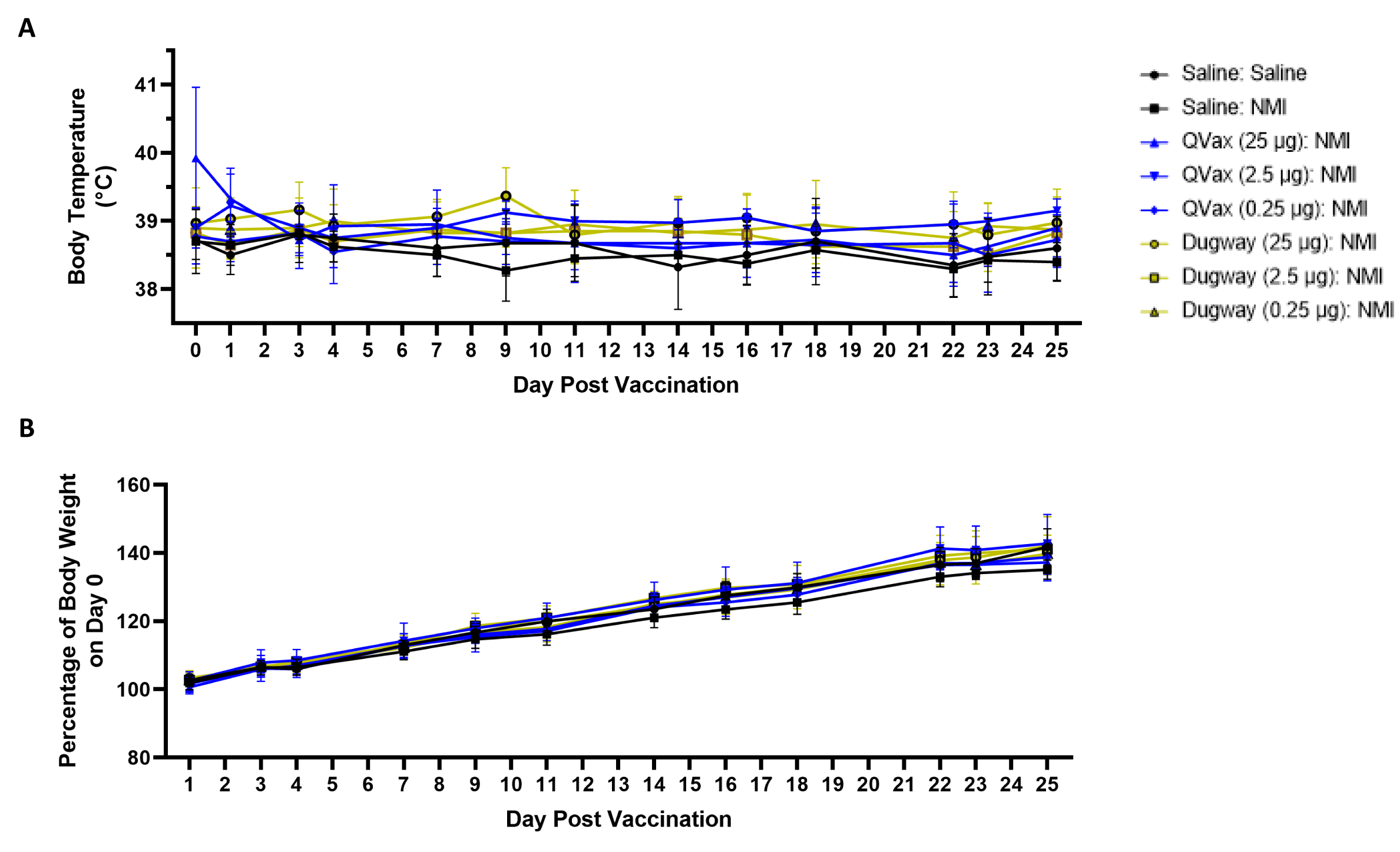

Supplement: Supplementary file 1 [file microorganisms-10-02261-s001.zip › Figure S3.tif]

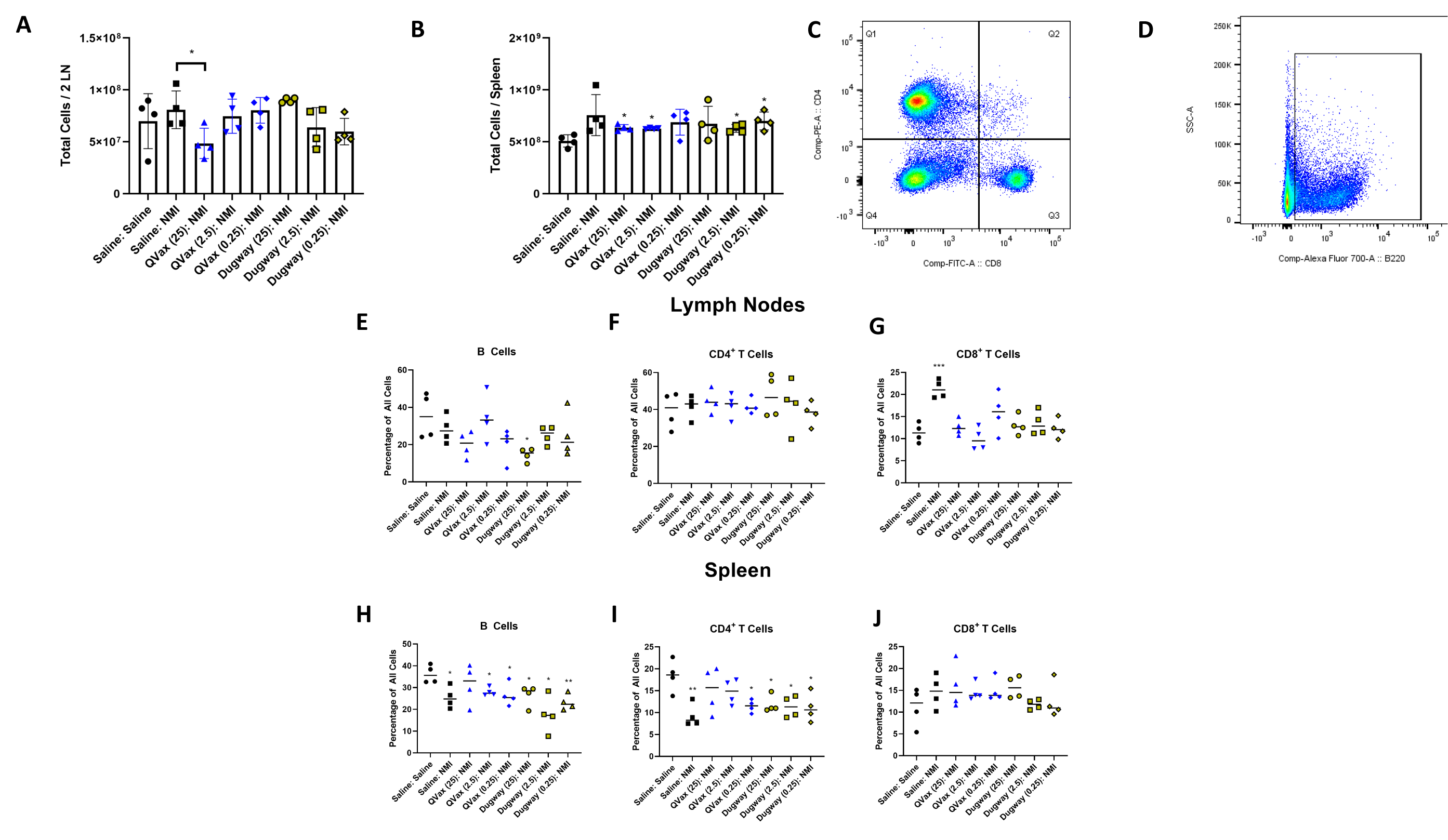

Supplement: Supplementary file 1 [file microorganisms-10-02261-s001.zip › Figure S4.tif]

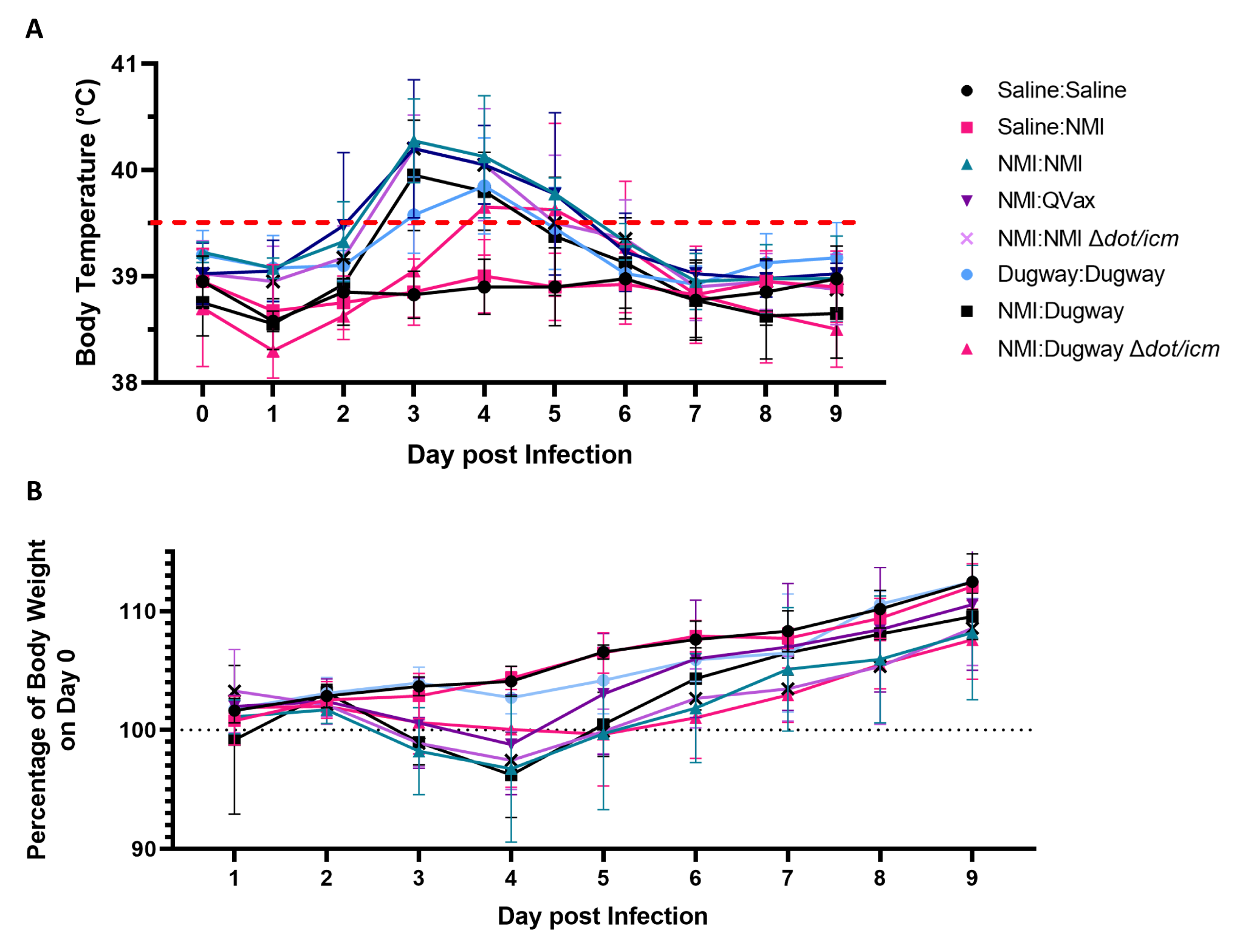

Supplement: Supplementary file 1 [file microorganisms-10-02261-s001.zip › Figure S5.tif]

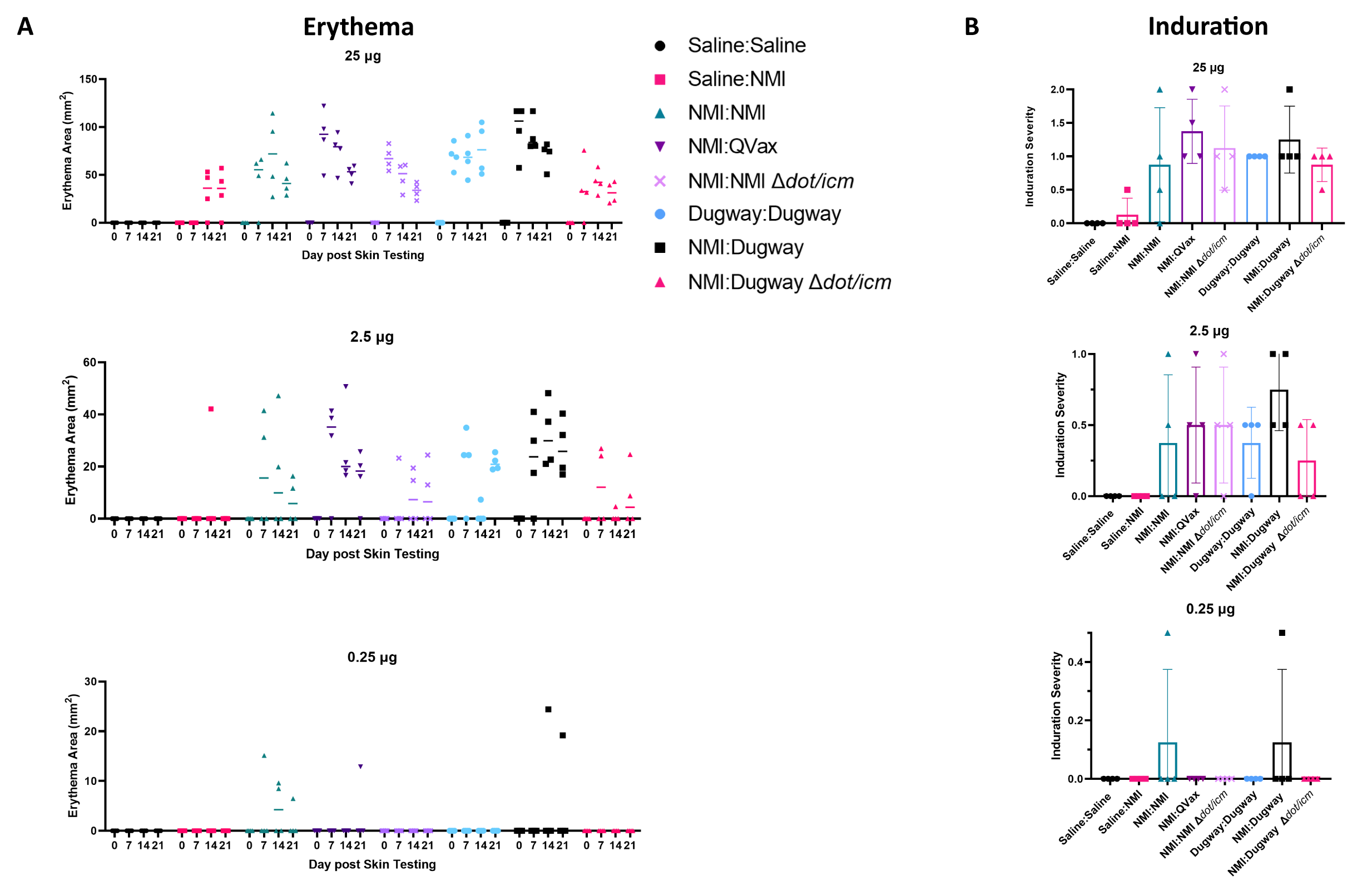

Supplement: Supplementary file 1 [file microorganisms-10-02261-s001.zip › Figure S6.tif]

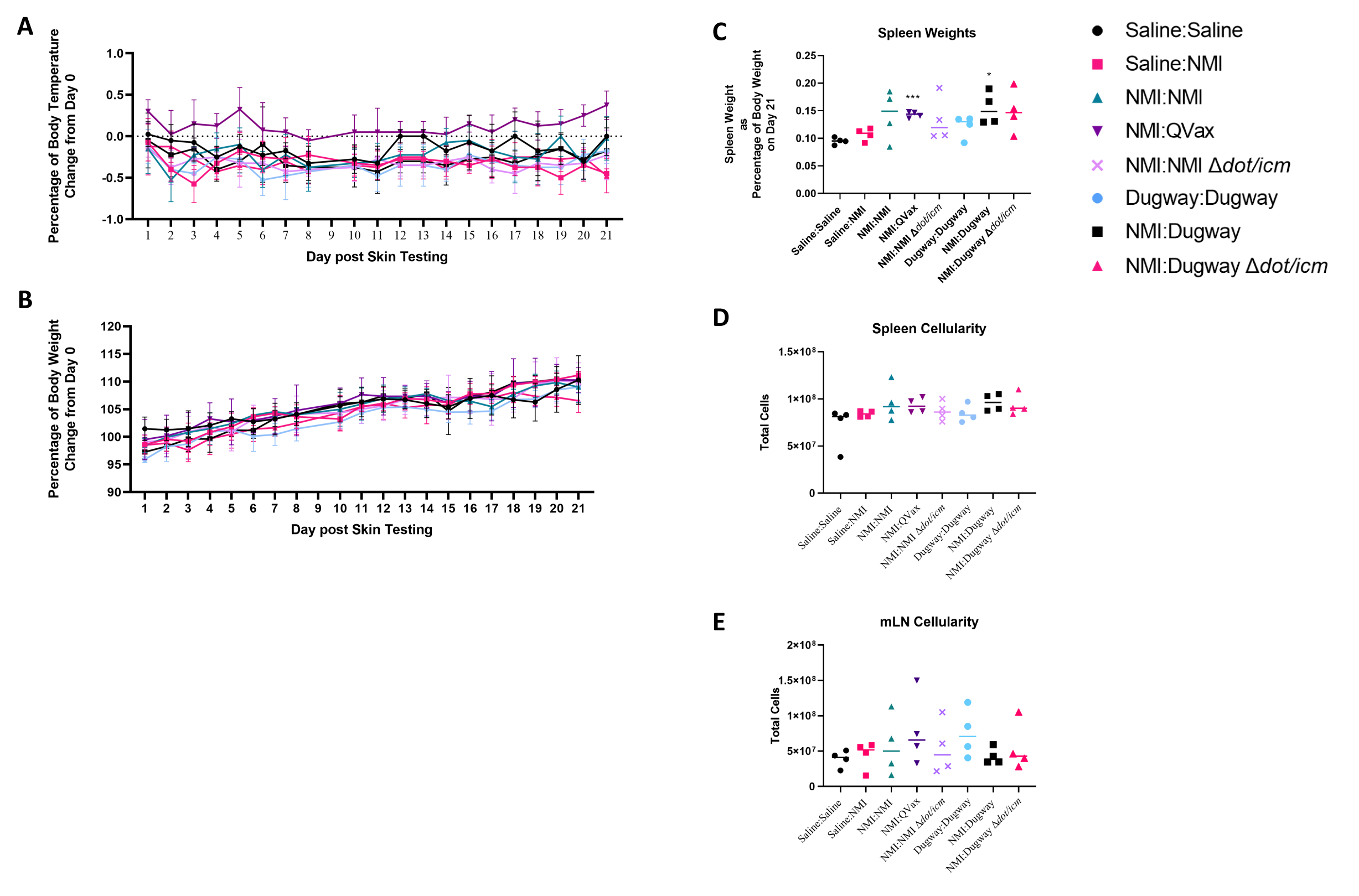

Supplement: Supplementary file 1 [file microorganisms-10-02261-s001.zip › Figure S7.tif]
